# Effectiveness of a Self-Fitting Tool for User-Driven Fitting of Hearing Aids

**DOI:** 10.3390/ijerph182010596

**Published:** 2021-10-10

**Authors:** Matjaž Debevc, Mark Žmavc, Michael Boretzki, Martina Schüpbach-Wolf, Hans-Ueli Roeck, Alamgir Khan, Andrew Koubatis, Sašo Jezernik, Ines Kožuh

**Affiliations:** 1 Faculty of Electrical Engineering and Computer Science, University of Maribor, 2000 Maribor, Slovenia; markzmavc@gmail.com (M.Ž.); ines.kozuh@um.si (I.K.); 2 Sonova AG, 8712 Stäfa, Switzerland; michael.boretzki@sonova.com (M.B.); martina.schuepbach-wolf@sonova.com (M.S.-W.); hans-ueli.roeck@sonova.com (H.-U.R.); 3 ALTRAN, 8005 Zürich, Switzerland; akh438@gmail.com (A.K.); andrew.koubatis@capgemini.com (A.K.); jezerniks@gmail.com (S.J.)

**Keywords:** hearing aids, assistive technology, hearing aid self-fitting, user-driven hearing aid fitting

## Abstract

Hearing aids can be effective devices to compensate for age- or non-age-related hearing losses. Their overall adoption in the affected population is still low, especially in underdeveloped countries in the subpopulation experiencing milder hearing loss. One of the major reasons for low adoption is the need for repeated complex fitting by professional audiologists, which is often not completed for various reasons. As a result, self-fitting procedures have been appearing as an alternative. Key open questions with these digital tools are linked to their effectiveness, utilized algorithms, and achievable end-results. A digital self-fitting prototype tool with a novel quick four-step fitting workflow was evaluated in a study on 19 individuals with moderate hearing loss. The tool was evaluated in a double-blinded, randomized study, having two study aims: comparing traditional audiological fitting with the new self-fitting tool, which can also be used as a remote tool. The main reported results show moderately high usability and user satisfaction obtained during self-fitting, and quasi-equivalence of the performance of the classical audiological fitting approach. The digital self-fitting tool enables multiple sessions and easy re-fitting, with the potential to outperform the classical fitting approach.

## 1. Introduction

Hearing impairment is a growing global issue for our society, especially among the elderly, and affordable hearing care is a major public health concern [1,2,3]. According to the World Health Organization (WHO), it was estimated that 466 million people worldwide (over 5% of the world’s population) have a disabling hearing loss, and it is predicted that this prevalence will triple by 2050 [4,5]. In Europe, around 117 million people have disabling hearing loss [6,7]. Sensorineural hearing impairment encompasses decreased sensitivity, dynamic range, frequency resolution, and temporal resolution. These elementary deficits are associated with speech and other kinds of sound being less intelligible and less easy to recognize, especially in noisy environments [8,9,10]. A loss of hearing is linked with numerous healthcare issues, such as depression, loneliness, cognitive decline, diabetes, cardiovascular disorders, and falls [11,12,13,14,15,16,17,18]. Compensating or mitigating the consequences of hearing impairment is the purpose of audiological solutions.

One way to improve hearing is the use of hearing aids (HA) with digital signal processing being worn either behind or in the ears. If correctly fitted (employing the correct setting of appropriate and adequate HA parameters to compensate for the hearing loss in the best possible way), an HA enables those with hearing impairment to listen, communicate, and participate effectively in daily activities [3,9,10,19,20].

According to a study performed in the United States, the overall satisfaction with hearing aids increased from 74% to 81% between 2008 and 2015 [19]. Critically, globally, only about 20% to 33% of individuals use a hearing aid [21]. Several studies have examined this question and found that the regular use of hearing aids has been low, with insufficient hearing improvements, and inappropriate fitting has been identified as one reason for this [22,23]. There are several reviews of the factors leading to low hearing aid adoption, for example, viewing the issue as a parameter-fitting procedural challenge [21,24,25,26].

The parameter-fitting (customization) procedure is typically performed by a trained audiologist. At audiology clinics audiologists often give a quick and general tutorial on the basic usage of the HA (that is their priority) and neglect the long-term skills such as cleaning the HAs, adjusting the program, and the use of the telephone [27]. If necessary, users can arrange another appointment for further instruction, but often without proper success [10,28,29,30]. Another challenge arose due to the COVID 19 pandemic restrictions whereby users had only the limited option of adjusting their HAs themselves with guided support at a distance [24].

Another reason for the HA fitting challenge is the growing number of different digital hearing instrument fitting processes, such as fitting for varied situations (speech in a quiet environment, loud environment, music, etc.), as well as the use of different compressions and algorithms, such as echo cancellation, noise reduction, and others [10,20,30]. Since there are so many different possibilities, audiologists and other hearing experts, who also use fitting software from various companies, may have some difficulties in fitting the HA (and in due time) to the needs of the end user [19,21,30].

### 1.1. Self-Fitting Systems

Recently, self-fitting systems have emerged and provided several advantages in terms of enhancing the fitting process [31]. Namely, HAs can be fitted without the participation of audiologists; users can operate with the HAs on their own; they can adjust amplification parameters and assess their hearing threshold, generally using smart devices such as smartphones and tablets [32,33,34,35,36].

One example of the self-fitting system has been presented in the Goldilocks software approach [34,37,38]. It was developed to allow users to operate with the parameters of sound fullness, loudness, and crispness. Their findings showed that the speech intelligibility index increased with self-adjustment to a level where at least 95% of words used in the study were recognized. Furthermore, the results of self-adjustment can approach those of widely used prescriptions in terms of the spectrum and output level. Similarly, a study by Liang, et al. [33] showed the importance of the user interface for gathering feedback from the user, which helps in parameter optimization and adjustment during the self-fitting process.

Existing research has already revealed the advantages and disadvantages of self-fitting HAs and smartphone-controlled HAs from the perspective of users [39,40].

Advantageously, the trial time of using the smartphone app improved the various benefits (increased communication ability and motivation in quieter environments, increased self-sufficiency) and reduced the noticeable barrier (hearing loss stigma) of using an HA [41]. Additionally, participants in another study noticed increased communication ability in different acoustic spaces and increased self-sufficiency [22].

However, one disadvantage encountered by elderly users of HAs is that some are presented with difficulties in using the technology [34,36,41]. They expressed the need for additional support for audiologists during fittings to provide reassurance [41]. Likewise, Perry, et al. [42] found that some different algorithms for self-fitting of the Ear Machine app may reduce speech intelligibility. Additionally, a study by Oliver, et al. [8] showed that the audiologist protocol gave better fitting results, based on the NAL-NL2 prescription procedure [43], than the two user-driven protocols.

According to the abovementioned studies [8,41,43], the use of smart devices for self-tuning is highlighted, although cautiousness is needed for the elderly, who are less efficient in the use of technology and some do not even use smartphones. Moreover, these studies have highlighted the factor of confidence in one’s ability to set the hearing aid to the optimal fitting value. Many users thus felt more confident if they were under control and were guided personally through the setting by someone, or at least checked to see if they had completed it successfully and if the setting was correct. There has also been a visible demand for an understandable, accessible, and intuitive user interface that would allow for a more successful user-driven protocol [8,41,42,43] This protocol should ideally be applicable for all types of hearing loss and all types of HAs.

### 1.2. Self-Fitting Prototype

To provide an alternative self-fitting approach and to achieve a complexity reduction with a level of simplicity as described in Valdez, et al. [44], an improved self-fitting user interface has been proposed to improve the visualization of the self-fitting process and the linking of a perceptual space to a simple mental metaphor, and to decrease the need for verbalizing auditory problems. This user interface will support the fitting of multi-program HAs which address different kinds of sound with different amplification settings. The user interface will also support the user in exploring the multitude of different amplification settings in an efficient and effective way. Additionally, the user interface will allow a pleasurable and intuitive user experience during fitting.

**Figure 1:**
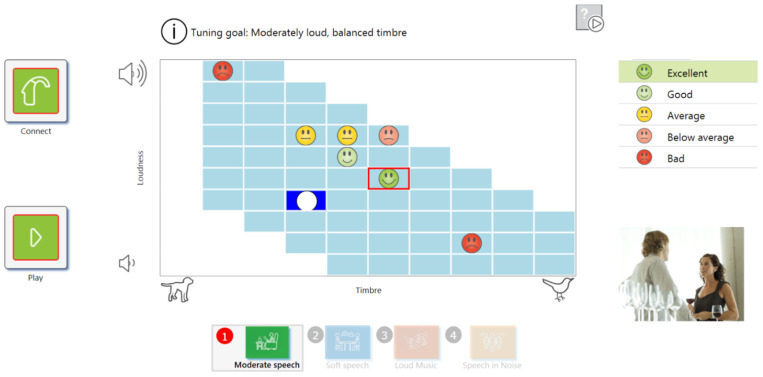
User interface of self-fitting system implemented in the fitting software.

The new system, the “Self-Fitting Prototype (SFP)”, built into a hearing aid manufacturer’s fitting software (Phonak TARGET), offers the possibility to complement the conventional fitting performed by the audiologist through self-fitting performed by HA users themselves. The user interface of the self-fitting process is shown in Figure 1.

At the top of the interface, users are given instructions about the ideal sound setting, which is to be found by exploring the matrix of cells. At the bottom of the user interface the SFP provides four sound environments, which enable self-fitting with four different kinds of sound: moderate speech, soft speech, loud music, speech in noise. The green buttons on the left are intended for enabling the HA connection and to play the sound of the current sound environment.

The main part of the user interface is a two-dimensional grid with cells for fitting the HAs. Vertically, the user can change the loudness, and horizontally the timbre. This is realized by varying the amplification settings. The amount of amplification is varied by selecting different cells in the vertical dimension. Moving upwards increases the amplification, and vice versa. Selecting different cells in the horizontal dimension changes the relation of high-frequency amplification to low-frequency amplification. Moving right increases the weight of high-frequency amplification and decreases low-frequency amplification.

After listening to the sound settings of a cell representing a certain fitting parameter, the users can evaluate each cell with one of five symbols (emoticons, as seen in Figure 1), marking their satisfaction with the cell’s setting for future reference. After finding the most suitable cell, the user exits the sound environment exercise with the green button on the right and goes onto the next exercise, with a different sound environment but the same user interface design and interaction.

The user interface provides the first step in fitting the aid. From moderate speech, the user is guided via a two-dimensional grid, enabling the adjustment of both the loudness and timbre of the sound to compensate for their hearing loss and thus to coarsely adjust the device to calibrate their individual spectral amplification needs.

The following three sound environments in the subsequent workflow steps (soft speech, loud music, and speech in noise) enable fine-tuning, by which the user tries to optimize the HA settings further.

The objective of the study was to examine the feasibility, benefits, and shortcomings of using a grid-based self-fitting tool for fitting HAs. The primary goal of the study was to collect initial feedback about the usability aspects [45] of SFP, to understand if users were able to satisfy their individual needs in several sound situations and to identify the potential problem areas. A secondary goal of the study was to assess the audiological impact of self-fitting compared to conventional fitting in terms of:

- Loudness comfort: No sound will be too loud.
- Sound familiarity: Hearing will not sound strange and should sound “natural”.
- Overall quality: Relevant sound stimuli will be audible, discriminable, localizable, and recognizable, as well as familiar and not too loud.

## 2. Methodology

### 2.1. Participants

Participants were recruited through advertisements in local associations for the hard of hearing, the database of a local audiology clinic, through invitation posters and leaflets given at the audiology clinic, social networks, and through the families and friends of colleagues of the local university.

The criteria for inclusion into the study were having mild-to-severe sensorineural or mixed hearing loss and being experienced in the use of hearing aids. The participants should be cognitively capable of understanding the instructions and questions of the study, and able to visit the study laboratory and other locations to test the hearing aids in the study. The exclusion criteria were mental illness, including high degrees of distress due to tinnitus [46,47,48,49], profound hearing loss, and sudden hearing loss [9,10]. The inclusion or exclusion of the participating candidates according to the said criteria was checked through personal interviews and with audiometric measurements.

Employing the abovementioned inclusion and exclusion criteria resulted in a study sample of 19 adults with permanent, stable hearing loss. The selected sample was sufficiently large for examining the initial usability of the system—not as a large-scale evaluation. With 19 participants we obtained sufficient data to show a clear first impression of the usability. In the event that the statistical analysis had not provided valid results, we would have invited more people. However, our study complies with the usual recommendations for usability studies [50].

Participants’ ages ranged between 20 and 73 years (M = 54.0 years, SD = 15.0 years), and they were well-balanced in terms of gender (10 females and 9 males). They had an average of 2.2 years of experience using HAs (SD = 1.9 years) and had been wearing HAs for at least a year in both ears, except for 2 participants who had only been using an HA in one ear. The reported duration of their hearing problems (M = 13.0 years with SD = 9.8 years) ranged from 3 to 32 years. The participants had a four-frequency average (4FA) hearing loss of M = 43.0 dB HL with SD = 5.8 dB HL, measured across 0.5, 1, 2, and 4 kHz.

Based on participants’ audiograms supplied before the study, the majority had binaural hearing loss. Three participants (16%) had no differences in the average hearing loss between the left and right ears, eight participants (42%) had up to 5 dB differences, six participants (32%) had 6–10 dB differences, and two participants (10%) had more than 10 dB difference, with 11 dB and 18 dB differences. Tinnitus was present in 42% of the participants. This heterogeneity of the sample was as expected in a random sample of hearing-impaired subjects, and contributed to the external validity of the study, as it reflects the variability of the population from which the sample was drawn [51,52,53,54].

The participants were asked to rate their experience with the technology using a 5-point scale from no experience to expert. On average, they were moderately experienced in technology use (M = 3.0 with SD = 1.3). Some reported having little to no experience with computers, smartphones, and tablets, whereas some considered themselves very experienced. None of them were technical experts in the sound amplification of HAs.

### 2.2. Materials

*Hearing Aids.* The study participants were fitted with a Phonak Audéo V90 312 with an xS receiver, which covers a range from 27 to 55 dB of hearing loss. The participants’ own hearing aids could not be used in the study as they were not compatible with the SFP. The hearing aids with AutoSense OS were programmed using the Phonak Adaptive Digital prescription formula. All hearing aids were fitted to ears with so-called “closed domes” [55], which are suitable for mild-to-moderate hearing losses.

*Twin Hearing Aids.* A system of switchable “twin hearing aids” was developed and used for the direct comparison of conventional and self-fitting results. Two agglutinated receiver-in-canal (RIC) hearing aids were mounted on each ear. They were connected to the receiver in the ear canal and to a switch which allowed them to choose a setting. The user could choose to listen through one or the other pair of hearing aids by operating a switch on the switch box. One pair of hearing aids was programmed with the result of the audiologist-driven (AD) fitting, the other one with the patient-driven (PD) fitting. The user could listen to an audio sample and answer a direct comparison question after having switched back and forth between listening through one or the other pair of hearing aids. For the purposes of blinding the subject and the experimenter, the hearing aid settings were labeled “red” and “green”.

*Booth.* The study was conducted in an acoustic modular booth with 180 cm width, 240 cm length and 205 cm height with a sound reduction value of 44 dB. A single loudspeaker was situated at ear height at 1.4 m distance in front of the participant. The sound presentation system was calibrated at the start of each test day.

*Audio samples.* To estimate the subjective qualities of each HA setting, users listened to 10 short audio samples in the same sequence: A very softly ticking clock, a soft flowing river, softly singing birds, a soft female voice, a moderate short dialogue, a moderate male voice, moderate classical music, a loud chirping cricket, loud classical music, and a very loud pneumatic hammer. These audio samples varied in sound pressure level from 34.5 dB to 85.3 dB. The examples were selected such that the audiological outcome of the fitting process could be evaluated by the subject for typical listening conditions (e.g., short moderately loud dialogue), rather difficult conditions (soft female voice), and rather uncomfortable conditions (e.g., the pneumatic hammer). The selection of audio samples was defined by the results of the study performed by Heller, et al. [56].

*Self-Fitting Prototype (SFP).* The user interface SFP, as seen in Figure 1, was given to the user. The SFP was running on a tablet PC.

### 2.3. Measures

The instruments intended to evaluate the usability aspects of the SFP were the User Experience Questionnaire (UEQ) [57], the quality assessment interview and the SFP-Questionnaire (Appendix A). The rating of audio samples, the environmental sounds questionnaire and the phonological speech test were used to evaluate the audiological outcomes of the SFP. We describe them briefly in the text below.

*User Experience Questionnaire (UEQ).* The UEQ is a 26-item questionnaire designed to capture the user’s experience during their interaction with the software. The UEQ contains six different user experience categories [58]—attractiveness, perspicuity, efficiency, dependability, stimulation, and novelty.

*Quality assessment interview.* A short interview was conducted after each participant’s use of the SFP to determine the user experience more precisely. It was used as the dominant qualitative user experience assessment method [59] to effectively elicit reactions regarding the participants’ engagement effectively [60]. We inquired about the strengths and weaknesses of the app, their perceptions of the learning process, ideas for app improvement, and whether any part of the self-fitting process was still unclear to them. Based on participants’ responses, further questions were sometimes asked to clarify their answers.

*SFP-Questionnaire.* Based on a preliminary study on SFP usability (*n* = 8), we wanted to address some usability concerns specific to the tool. Since the UEQ only addresses general usability issues, we developed an SFP-Questionnaire (Appendix A). Four of the 12 questions referred to the participant’s ability to discriminate the loudness or frequency of the sounds of different cells. An additional 4 questions asked about difficulties in evaluating sounds and making decisions based on those evaluations. The rest of the questions addressed the extent of mental effort invested and the user’s confidence in the final cell choices. As this is a self-reported measure, we acknowledge the possibility of overestimating one’s own abilities.

*Perceptual rating of audio samples.* This was used as it plays an important role in a wide range of research on audio quality [61]. Users evaluated audio samples, presented on a touchscreen, in terms of their loudness, familiarity, and overall quality on a 9-step rating scale. Each sample was presented at the same sound level to all participants and in the same sequence. When assessing sound familiarity, only 4 samples were used (speech and music samples), as some participants may not have been familiar with the other sounds.

*Comparative perceptual rating of audio samples.* Using twin HAs and a switchbox (see the Materials section) participants compared the two HA settings directly. Using a comparative 9-step rating scale they reported whether loudness, familiarity, and overall quality of the “red” or “green” setting was higher for each of the audio samples.

*Environmental sounds questionnaire.* Participants were asked to listen to surrounding sounds as they walked around in the vicinity of the lab, in the building and outside for 20 min, with the new HA setting. They were asked to pay attention to male, female, and children’s voices, any music they might hear, their perception of the surrounding noisy and quieter environments. Afterwards, they filled in a questionnaire, evaluating those sound experiences. Note that the uncontrolled nature of the environmental sounds does decrease the reliability of the HA settings’ evaluations by users. Such an approach upgraded the methodological approach successfully implemented in previous research [62], where individual differences and preferences for environmental sounds were measured.

*Phonological speech test.* A series of 20 monosyllables was played from the speaker at a 60-dB sound level, measured at a distance of 1.4 m from the speaker. Participants were asked to repeat each monosyllable immediately, exactly as heard. The researcher noted whether each participant’s reply was correct or incorrect. Based on this response, the number of correctly recognized words was calculated. The methodological approach of the phonological speech test derived from the recommendations for the constructions of multilingual speech test [63].

*Researcher observations.* One researcher was tasked with observing and recording all user comments, complaints, and behavior during self-fitting to provide a log of user behavior and experience as a source for further research and development [50]. The list of observations was later re-examined and supplemented by reviewing the video footage of the entire self-fitting process.

### 2.4. Procedure

Each participant was invited to the lab for two sessions, approximately one week apart. The first session consisted of three parts, separated with 20-min breaks (Table 1):

- Audiologist-driven (AD) fitting using the Phonak Target application and its evaluation.
- Patient-driven (PD) fitting of hearing aids using the SFP and its evaluation.
- First direct comparison between AD and PD settings using comparative perceptual ratings of audio samples.

Table 1 shows which measures were applied at each stage of the procedure. The UEQ, Quality assessment interview, and SFP-Questionnaire were only applied to the PD fitting parts of the procedure as the AD fitting does not include a fitting action by the user.

## 3. Results

### 3.1. Usability Evaluation

First, we report on the general usability results as gauged by the UEQ. The average category results across the 19 participants are reported in Table 2. Based on their feedback after the first use, the attractiveness and novelty of the app were categorized as excellent (above the 90th percentile in a dataset of 401 products). SF’s stimulating capacity was also evaluated as excellent (above the 90th percentile), which means that users felt mentally engaged by the app and enjoyed using it. The dimension of perspicuity, indicating the ease of learning, was evaluated as good (between the 75th and 90th percentiles), as was the dimension of efficiency. Lastly, app dependability was above average (between the 50th and 75th percentiles).

Aiming to investigate the usability results further, we present a brief and selective summary of the quality assessment interview. The bulk of the relevant information was given as answers about the positive and negative aspects of the app. Regarding positive aspects, there was a clear pattern of users valuing the idea of self-fitting without an audiologist’s help, as 14 out of 19 (74%) participants mentioned this explicitly as a positive aspect. A related benefit, mentioned by five (26%) participants, was adjusting settings according to their current environment. One user did not report any positive app qualities. Regarding negative aspects of the app, six (32%) users mentioned having trouble in the learning process—some elements of the app were counterintuitive or not understood. Secondly, issues with the quickness and smoothness of the app due to slow response and loading times were present for five (26%) participants. An additional five (26%) participants did not report any negative app qualities.

The next group of specified usability feedback was obtained from the answers to the SFP-Questionnaire (Appendix A). Four items referred to the participant’s susceptibility to sound differences between cells of the Graphical User Interface in terms of loudness and frequency. Overall, participants were quite confident in their ability to differentiate between sounds, with 89% of responses being: I could tell the difference “almost always” or “often”, whereas 7% of replies were “sometimes” and 4% “rarely”.

In the process of evaluating sounds during the PD fitting, according to the SFP-Questionnaire, many participants (53%) found it somewhat difficult to determine whether they liked a particular sound setting or not, whereas the remainder found it relatively easy or very easy. Moreover, determining why a certain sound was disliked was sometimes relatively difficult (15%), or somewhat difficult (15%), although it was easier for the rest. When asked about confidence in their final cell choices, 53% had slight doubts about making ideal choices, and 47% thought they chose optimally.

In terms of mental effort during PD fitting, the majority of the participants reported that the tasks required some effort or minimal effort (73%), with only one person reporting a significant amount of effort required, according to the SFP-Questionnaire. Memorizing the sounds of previously tried cells was mostly relatively easy (53%) or somewhat difficult (32%). Most participants decided on the next cell choice with relative ease (82%), while also not choosing the next cells randomly (79%).

The final group of findings about usability originated from researcher observations (see Measures above). Eighteen out of 19 participants had comments and questions during the short tutorial phase and required explanations from the audiologist. Ten participants required an additional explanation about the functionality of the tuning grid and its two axes. After all direction was provided and self-fitting began, some misunderstandings and small issues were still present, but did not seem to endanger self-fitting completion for most participants. Seven participants were reluctant to listen to sounds on the extremes of each dimension, whereas five participants thought the starting (middle) cell represented an ideal setting. Nine participants needed encouragement to try more cells, and six participants commented on long cell response times. In summary, learning the functionality of the SFP took some effort and detailed instruction, whereas in the self-fitting process, users seemed more confident and relatively effective, completing the exercises with little outside help.

### 3.2. Evaluation of the Audiological Outcomes of Self-Fitting

**Figure 2:**
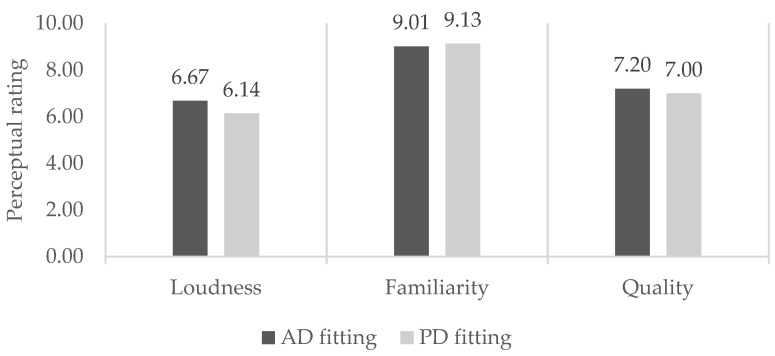
A visual representation of the perceptual ratings of audio samples at PD and AD settings (Mean values).

The perceptual ratings of loudness, familiarity, and the quality of sounds with PD settings and AD settings are reported in Table 3. The Kolmogorov–Smirnov test results indicated that the data deviated significantly from normal distributions, which is why nonparametric statistical procedures were implemented (the Wilcoxon signed ranks test). The only significant difference between the two fittings was in terms of the perceived loudness of sounds, where, on average, the AD setting was perceived as louder. The slight differences between settings in terms of perceived sound familiarity and overall quality were not statistically significant. The data visualization in Figure 2 shows the small rating differences between fittings, relative to the scale.

The first and second comparative perceptual ratings of the loudness, familiarity, and quality of sounds with PD and AD settings are reported in Table 4. Again, the Kolmogorov–Smirnov test results showed deviation of the data from a normal distribution, which led to the use of a one-sample Wilcoxon signed ranks test.

When sound settings were compared directly, no category evaluations yielded a significantly different result. However, a slight pull toward higher loudness, familiarity, and quality scores of AD settings was apparent, as is clear from the exclusively negative Mean scores shown in Table 4. Consistently with the indirect comparisons, the difference in the loudness of sounds was larger than in the other two categories.

The comparisons between environmental sound evaluations of PD and AD hearing aid settings are reported in Table 5. We analyzed and presented the evaluation of three categories: quiet environment, female voices, and male voices. Other categories were excluded from the analyses due to many missing values (e.g., the music category), or due to their consisting of only one value (e.g., the noise category). Similarly to previous analyses, we opted for a Wilcoxon signed ranks test due to deviations from a normal distribution. We found no statistically significant differences between PD and AD settings in evaluations of the three categories of environmental sounds. However, it should be noted that the small rating differences were consistently in favor of the AD setting.

Additionally, we present the average proportions of correct answers to the phonological speech test for each method. The AD fitting scored slightly better; users averaged 87.1% correctly replicated monosyllables, whereas the PD fitting resulted in 80.8% correct answers.

## 4. Discussion

The goal of this study was to determine the viability of a tool with a grid-based interface (with loudness and timbre as the two dimensions) as a means of the independent fitting of hearing instruments by users themselves. In terms of its usability, the primary focus of the study, the SFP fitting approach proved to be usable and effective overall; according to researcher observations, all participants were able to complete the self-fitting procedure after they were properly instructed on how the tool worked. According to the UEQ, participants found the app stimulating, novel, and attractive, as they reported appreciation for the potential benefits of fitting with no outside help. Interestingly, they did not report having trouble discriminating between the subtle differences in sound experiences of adjacent cells and did not perceive the self-fitting procedure as mentally demanding.

This is a remarkable difference compared to conventional fitting. In an AD fitting the user is not educated to do the fitting actively him/herself. Instead, the user answers questions from the audiologists about perception while the audiologist operates the fitting software to adjust the hearing aid settings based on the descriptions of the user. The PD fitting involves the user more actively in the fitting process, in order to better adjust the hearing aids to the users’ hearing needs.

However, some aspects of the SFP’s usability require improvement. The process of learning how the tool works was commonly lengthy according to researcher observations. The short tutorial that had been designed did not result in a sufficient level of understanding, as some aspects of the tool (e.g., the grid axes), were still misunderstood. Optimizing the learning curve and the intuitiveness of all tool features is an important issue moving forward, as many, primarily elder HA users, are not particularly experienced with computers, tablets, and smartphones.

The second usability aspect, which users were not satisfied with, was some lack of smoothness and the response time of the app, with cell response times being the main issue. Considering that the process of HA fitting typically requires establishing different levels of amplification across a frequency range, it is reasonable to expect the fitting tool to contain features and algorithms which are relatively complex. This can influence the app’s responsiveness negatively or increase the incidence of bugs, decreasing the perceived dependability and effectiveness of the tool. Conceptual simplicity in self-fitting tools of the future is, therefore, desirable.

A third, potentially challenging, usability aspect of patient-driven fitting may be its complete dependence on the person’s experience as opposed to the audiologist-driven fitting, which is based largely on the hearing loss data and audiological models and expertise on how to adjust the sound for mitigation of the consequences of hearing loss. Indeed, many participants reported they had some trouble deciding whether they liked a certain sound experience (53%), whereas some had trouble establishing what exactly was the reason (30%). Understandably, 53% of our sample had slight doubts about their final choices of HA settings. Considering that the self-fitting process was a complete novelty to the participants, and that they did not fully trust their adjustments, this outcome might be expected.

The secondary goal of the study was to compare the audiological outcomes of the self-fitting process versus the conventional audiologist-driven fitting process. Participants’ ratings revealed the perceived loudness, familiarity, and quality of the two HA settings to be similar, as the average differences were categorically less than 1 unit on a 9-step Likert scale. The only significant rating difference was in the loudness category, in which the AD setting was evaluated consistently as louder. Although we do not report the HA amplification data explicitly, it is likely that the AD setting, on average, provided more gain, as was the case in Boymans and Dreschler [39]. Future endeavors to explore the possibilities of avoiding the described usability shortcomings would, therefore, certainly be valuable to hearing aid users.

## 5. Conclusions

We have substantiated that the self-fitting approach based on a grid interface is a reasonable alternative to the audiologist-driven fitting approach, both in terms of its usability and audiological outcomes. It promises to be an addition to conventional HA fitting, and a convenient solution, providing more freedom in personal preferences in adjustments, not depending on additional visits to audiologists. The study also highlighted areas of possible improvement, where the learning process, responsiveness of the app, and user confidence should be given additional attention in future endeavors. As an argument against these shortcomings, we emphasize that only one iteration of self-fitting was performed in the study, whereas, in the intended use of the app, users would be able to complete multiple iterations of the self-fitting procedure. As users improve their mastery of the functionality of the app and grow to become confident in self-fitting through continued use of the tool, we expect them to work towards even better audiological outcomes and satisfaction with hearing aids.

Additionally, more research on the viability of self-fitting tools with a larger sample size would be welcome, in order to establish the challenges of this approach accurately. Importantly, both quantitative, as well as qualitative research methods should be employed, considering the novelty and complexity of the self-fitting process.

## Figures and Tables

**Table 1:** The procedure of the first session for each participant group.

	Group 1 (*n* = 10)	Group 2 (*n* = 9)
Part 1	PD fitting with SFP SFP-Questionnaire User Experience Questionnaire (UEQ) Quality assessment interview Perceptual rating of audio samples Phonological Speech Test	AD fitting with Phonak Target software Perceptual rating of audio samples Phonological Speech Test
Break	Stroll outside + Environmental sounds questionnaire upon return	Stroll outside + Environmental sounds questionnaire upon return
Part 2	AD fitting with Phonak Target software Perceptual rating of audio samples Phonological Speech Test	PD fitting with SFP SFP-Questionnaire User Experience Questionnaire (UEQ) Quality assessment interview Perceptual rating of audio samples Phonological Speech Test
Break	Stroll outside + Environmental sounds questionnaire upon return	Stroll outside + Environmental sounds questionnaire upon return
Part 3	Comparative perceptual rating of audio samples	Comparative perceptual rating of audio samples

**Table 2:** Usability aspects of the self-fitting prototype in comparison with other products.

Scale	Mean ^1^	Interpretation
Attractiveness	2.18	In the range of the 10% best results
Perspicuity	1.84	10% of results better, 75% of results worse
Efficiency	1.68	10% of results better, 75% of results worse
Dependability	1.42	25% of results better, 50% of results worse
Stimulation	1.91	In the range of the 10% best results
Novelty	1.64	In the range of the 10% best results

^1^ Scored on a scale from −3 to 3.

**Table 3:** Perceptual rating of audio samples with PD and AD settings.

	Items	Mean (AD) ^1^	Mean (PD) ^1^	Z	Sig.
Loudness	10	6.674	6.137	−2.269	0.023
Familiarity	4	9.012	9.132	−0.917	0.359
Quality	10	7.200	7.000	−0.443	0.658

^1^ Scored on a scale from 1 to 10.

**Table 4:** Comparative perceptual rating of audio samples between PD and AD settings.

	Items	Mean ^1^	Z	Sig.
Loudness comp. 1	10	−0.640	−1.691	0.091
Familiarity comp. 1	4	−0.342	−1.182	0.237
Quality comp. 1	10	−0.071	−0.308	0.758
Loudness comp. 2	10	−0.282	−0.719	0.472
Familiarity comp. 2	4	−0.224	−0.544	0.586
Quality comp. 2	10	−0.267	−0.697	0.486

^1^ Scored on a scale from −4 to 4. Negative values indicate a preference for AD settings.

**Table 5:** Environmental sound evaluation comparison between PD and AD settings.

	Items	Mean (AD) ^1^	Mean (PD) ^1^	Z	Sig.
Quiet environment	3	7.316	7.130	−0.385	0.700
Female voices	5	7.053	6.842	−0.566	0.571
Male voices	5	7.242	6.905	−1.041	0.298

^1^ Scored on a scale from 0 to 8. Higher scores indicate a better evaluation.

## Data Availability

The raw data supporting the conclusions of this article are freely available on https://github.com/debevc/tuneperfect/, (accessed on 1 August 2021).

## Appendix A

**Table A1:** Self-Fitting Prototype (SFP) Questionnaire.

How often did you feel the difference between the sounds of adjacent cells?	
(a)	Almost never
(b)	Rarely
(c)	Sometimes
(d)	Often
(e)	Almost always
How often did you feel the difference between the sounds on opposite sides of the grid?	
(a)	Almost never
(b)	Rarely
(c)	Sometimes
(d)	Often
(e)	Almost always
When moving up and down the grid, how often did you feel the difference in sound level?	
(a)	Almost never
(b)	Rarely
(c)	Sometimes
(d)	Often
(e)	Almost always
When moving left and right in the grid, how often did you feel the difference in clarity and fullness of sounds?	
(a)	Almost never
(b)	Rarely
(c)	Sometimes
(d)	Often
(e)	Almost always
How difficult was it to decide whether you liked a sound or not?	
(a)	Very easy
(b)	Relatively easy
(c)	Somewhat difficult
(d)	Relatively difficult
(e)	Very difficult
How hard was it to determine why you disliked a certain sound?	
(a)	Very easy
(b)	Relatively easy
(c)	Somewhat difficult
(d)	Relatively difficult
(e)	Very difficult
How difficult was it to decide which cell to try next?	
(a)	Very easy
(b)	Relatively easy
(c)	Somewhat difficult
(d)	Relatively difficult
(e)	Very difficult
How did you decide which cell to try next?	
(a)	I mostly chose randomly.
(b)	I mostly chose a cell in the direction of the desired sound change.
How difficult was it to remember what previously tried cells sounded like?	
(a)	Very easy
(b)	Relatively easy
(c)	Somewhat difficult
(d)	Relatively difficult
(e)	Very difficult
How much mental effort did the process of choosing an ideal cell require?	
(a)	Minimal effort
(b)	Some effort, but not a lot
(c)	Sometimes little, sometimes a lot
(d)	A significant amount of effort
(e)	A lot of effort
How certain are you about choosing ideal cells for you?	
(a)	I am very doubtful
(b)	I am slightly doubtful
(c)	I am certain
How would you evaluate the final sound settings of your chosen cells?	
(a)	Mostly bad.
(b)	Bad more often than good.
(c)	Sometimes bad, sometimes good.
(d)	Good more often than bad.
(e)	Mostly good.
